# The use of psychological interventions in tertiary prevention programs for individuals engaged in violent extremism: a scoping review and interviews

**DOI:** 10.1186/s40352-025-00324-w

**Published:** 2025-03-22

**Authors:** Daisy Muibu, Anna Vasaturo, Wilson Spurrell, Elena Savoia

**Affiliations:** 1 Harvard T.H. Chan School of Public Health, Harvard University, Boston, USA; 2 Department of Defense, Africa Center for Strategic Studies, Washington D.C., USA; 3 Northeastern University, Boston, USA

**Keywords:** Terrorism, Extremism, Deradicalization, Reintegration, Tertiary Interventions, Psychological Intervention

## Abstract

Psychological interventions are used as part of tertiary countering violent extremism (CVE) efforts throughout the world, but it remains unclear the type of psychological treatments commonly used as part of such interventions. This study combines a scoping literature review with interviews to identify and describe the type of psychological treatments used, as well as their structure, and implementation. The scoping review identified 34 tertiary prevention programs that include a psychological intervention spanning 22 countries. Additionally, 18 interviews with mental health providers and other practitioners directly involved in delivering tertiary prevention programs allowed us to complement the results of the literature review.

**Supplementary Information:**

The online version contains supplementary material available at 10.1186/s40352-025-00324-w.

## Background

In the field of countering violent extremism (CVE), the aim of tertiary prevention programs is to disengage the individual from a violent extremist group and/or from violent or criminal behavior associated with violent extremism, and in some cases encourage the individual to abandon their extremist identity. In other words, tertiary prevention programs aim to “*specifically target individuals who are already associated with violent extremist groups or who directly participate in this violence*” (Khalil et al., 2019b, pg. 444).^1^ Such tertiary prevention programs can also involve the reintegration of individuals following incarceration or participation in an extremist group or cause. In some cases, these programs are implemented in prison settings or rehabilitation centers and often involve the provision of basic education, vocational training, and psychological and social support, among other services.

It is worth noting that, in the violent extremism literature, terminology such as “deradicalization,” “disengagement”, and “reintegration,” are often used interchangeably and inconsistently to describe such programs (Glazzard, 2022; Koehler, 2020). In this manuscript, we will refer to tertiary prevention programs that focus on any of these three approaches as defined in Table 1.

**Table 1:** Definitions (verbatim as needed)

Deradicalization	The cognitive process through which an individual abandons their extremist identity and worldview. This generally involves an attitudinal change. (Horgan & Braddock, 2010; Schuurman & Bakker, 2015)
Disengagement	The behavioral change whereby individuals withdraw from violent behavior and/or a group involved in violent extremism (Horgan & Braddock, 2010; Reinares, 2011)
Reintegration	The re-establishment of positive social, economic, familial, and community ties (Holmer & Shtuni, 2017)

Despite an anticipated need to increase the development of tertiary prevention programs for individuals with a history of involvement in violent extremism, documentation on the use of specific psychological interventions as part of these programs remains rare, with limited data not only on their effectiveness (Rousseau et al., 2021a, 2021b) but also on what specific techniques and approaches are being used. By “psychological intervention,” we refer to any therapeutic technique used to promote mental well-being or treat mental disorders, including any type of individual or group therapy, psychoeducation, and cognitive, behavioral, or emotional skills training.

The association between mental health issues and violent extremism remains a controversial topic, and research suggests a distinction between those who are lone actors and those who are members of extremist groups. Based on available evidence, overall engagement with an extremist group has not been found to be causally linked to psychiatric problems (Rousseau et al., 2022). In fact, Gill et al., (2021, p. 66) and Misiak et al. and’s (2019, p. 56) systematic reviews examining this relationship both concluded that a “unique profile of psychopathology or personality traits that makes individuals more prone to radicalization cannot be proposed based on available evidence.” However, there appears to be a distinction between lone and group extremists, with lone actors being more likely to present with mental health conditions (Corner & Gill, 2015; Corner et al., 2016). Gruenewald et al. (2013) found differences in the prevalence of mental health diagnoses between members of U.S.-based far-right terrorist groups and far-right lone actors, with 7.5% of the former (group terrorists) having a confirmed mental health diagnosis, compared to 40.4% of the latter (lone-actor terrorists). Similarly, Gill et al.'s (2021) systematic review, among other things, concluded that lone- and group terrorists have two distinct profiles in terms of their drivers, criminogenic needs, and psychopathology.

It is too soon to presume a causal relationship between mental disorders and lone actor attacks, and to do so would run the risk of stigmatizing persons with mental illness and “amalgamating social and political dissent with mental illness and criminal tendencies” (Rousseau et al., 2022, p. 2). There exist multiple pathways into violent extremism and many factors can contribute to a single individual’s pathway. In fact, “rarely are mental health problems the sole issue” that contributes to the risk of an individual engaging in extremist behavior (Gill et al., 2021, p. 67). Yet, for lone actors and other extremists that do present psychiatric problems, psychological interventions may offer a more effective risk reduction strategy than security-focused approaches, such as incarceration, that dominate the fields of counterterrorism and violent extremism prevention (Hassan et al., 2021; Rousseau et al., 2022).

Existing systematic reviews and meta-analyses on the subject of psychological intervention used as part of tertiary programs offer some insights into the potential benefits of such interventions. For instance, a review of tertiary prevention programs for individuals released from jail suggests that interventions that include psychosocial counseling and therapy report more positive outcomes, such as the adoption of healthy coping strategies, improved psychological health, and the lifting of the psychological burden that ideological exploitation and coercion placed on them, among other outcomes, compared to those who do not include such approaches (Hassan et al., 2021). Moreover, an evaluation of CVE interventions in Australia highlights the positive impact that programs with psychological and counseling services have on disengagement (Cherney & Belton, 2021). Similarly, Jugl et al.’s (2020) meta-analysis of outcome evaluations of psychosocial prevention programs implemented in several countries found positive effects on both behavioral and psychosocial outcomes (including attitudes) related to extremism.

Despite this emerging literature, most of which focuses on the impact of these interventions, there remains a gap in the documentation of what type of psychological interventions have been implemented in tertiary efforts in the first place. To fill this gap, we combined the results of a scoping literature review with interviews with practitioners engaged in reintegration efforts.

## Methods

The question this study seeks to answer is: *What psychological interventions have been used in tertiary programs for the deradicalization, disengagement, and reintegration of extremists?* This methodology consists of 1) a scoping review of scientific and gray literature to identify tertiary prevention programs that include a psychological intervention and, 2) interviews with professionals experienced in the design and/or delivery of psychological interventions as part of tertiary prevention programs.

### Scoping literature review of tertiary programs including a psychological intervention

This literature review was conducted following the Preferred Reporting Items for Systematic Reviews and Meta-Analyses (PRISMA) statement (Page et al., 2021). Below we describe our search strategy, eligibility criteria, extraction, and synthesis methods.

#### Search Strategy

We conducted our search across a broad array of databases that covered several relevant disciplines, including political science, sociology, criminology, and psychology. These databases are: Sociological Abstract; socINDEX (EBSCO); Criminal Justice Abstracts; Web of Science; Proquest Social Science Premium Collection; EBSCOhost Academic Search Premier; Scopus; Pubmed; Medline; and PyscInfo. The search strategy included the following search strings:

Ab (psycho* OR "mental health" OR therap* OR intervention OR rehabilitat* OR treat*) AND ab(terroris* OR extremis* OR "violent extremis*") AND ab(radical* OR "targeted violence" OR "counter-terrorism" OR mobili* OR "countering violent extremis" OR disengag* OR deradicali* OR demobili* OR reintegrat* )

OR

ti(psycho* OR "mental health" OR therap* OR intervention OR rehabilitat* OR treat*) AND ti(terroris* OR extremis* OR "violent extremis*") AND ti(radical* OR "targeted violence" OR "counter-terrorism" OR mobili* OR "countering violent extremis" OR disengag* OR deradicali* OR demobili* OR reintegrat* )

Articles were limited to the English language and published prior to December of 2022. In addition to collecting scientific articles, we also conducted a search for gray literature on the topic. To identify gray literature sources, we relied on the Luxembourg’s definition of gray literature as that which is “produced on all levels of government, academics, business and industry in print and electronic formats, but which is not controlled by commercial publishers i.e., where publishing is not the primary activity of the producing body.” (Schöpfel, 2010) and consulted public inventories of institutions in the field of terrorism and counter-terrorism research (Bergeman & Kearney, 2021).

In addition, we consulted with six experts in the field, who shared gray literature reports, and suggested the websites of organizations and centers where we could look for additional information. In total, we compiled and searched through the websites of 61 institutions/organizations as listed in the Appendix section I.

#### Eligibility criteria

We retrieved articles that describe existing or previously existing tertiary prevention programs that include a psychological intervention. This psychological intervention did not have to be the primary focus of the article for the program to be included in our list. Editorials discussing conceptual or theoretical aspects of such interventions without referring to a specific program were excluded. Moreover, due to difficulty with access, we did not review books as part of this review. Accordingly, our review includes articles and reports about existing or previously existing tertiary prevention programs that involve some type of psychological intervention.

#### Reviewing and extraction process

We exported the articles as RIS files and uploaded them to Covidence – a primary screening and data extraction tool used to organize and streamline the process of conducting a systematic review (Covidence). Each article was screened by two researchers independently. A third researcher was consulted to resolve any discrepancies. For the articles that made it through the initial abstract and title screening, we then uploaded the full text onto Covidence and proceeded with the full text review, which was conducted independently by two reviewers.

Subsequently, we utilized data extraction coding procedures which involved identifying: 1) the psychological intervention; and 2) the setting of the program. The coding sheet used to describe the psychological intervention captured the following information: 1) the specific psychological treatments used with beneficiaries; 2) the type of personnel involved in delivering the psychological treatment (e.g., licensed clinicians, social workers, civil society actors/mentors, former extremists etc.); 3) the duration of the psychological treatment; 4) whether the psychological treatment was delivered in group or individual sessions; and 5) the level of risk participants posed when entering the program.

When coding for the setting in which the psychological intervention is administered, we noted: a) whether the program was run by a government agency; b) whether the program is custody/detention/prison-based (hereafter referred to as custody-based); and c) whether participation was voluntary or not.

We utilized a similar process to code gray literature materials. However, we did not use Covidence and rather than review abstracts and titles for the initial screening, we looked at the reports’ executive summaries and titles or skimmed the entire report. We used a spreadsheet to track our progress, with two reviewers independently screening the reports with a third reviewer called upon when necessary.

### Interviews with professionals with experience in tertiary programs

#### Interviewees and sample

We contacted 72 individuals with experience and expertise in CVE programming. Interviewees were selected based on an initial list of contacts from previous projects and expanded through snowball sampling (Naderifar, 2023) to purposefully identify professionals working across a range of extremist ideologies and professional contexts. Sampling concluded when we achieved saturation of content, defined as reaching a state of repetition of the information being gathered. The interview protocol was approved by the Harvard T. H. Chan School of Public Health Institutional Review Board (IRB). Every interview participant was sent a consent form and was asked to confirm their consent to participate by e-mail.

Out of the 72 contacted, 26 experts agreed to be interviewed, and among this sample, 18 had experience with delivering tertiary programs for violent extremists that included a psychological intervention. The 18 interviewees were from the following countries and worked primarily on interventions within that country: US (*n* = 13), Canada (*n* = 3), UK (*n* = 1), Lebanon (*n* = 1). In terms of professional profile our sample included: licensed mental health providers (*n* = 8), law enforcement officers (*n* = 3), social worker (*n* = 1), mentors/coaches (*n* = 3), researchers with experience in reintegration and program manager/developers (*n* = 3). It is worth noting that the preponderance of United States based interviewees was due to the need to gather information that could inform the development of programs in the US as required by the sponsor of this project.

#### Interview methods and analysis

We developed an interview guide to gather information on the psychological interventions being implemented in the tertiary prevention programs, type of professional figures engaged in the programs, and structure of the interventions (duration, group versus individual therapy). The interviews were conducted via Zoom in 2023 and lasted between 45–60 min each. All interviews were recorded and transcribed verbatim. One member of the team watched all the recordings to familiarize herself with the content of the interviews and selected those with sufficient information on the study question to be included in this analysis. Nvivo14, a software that facilitates qualitative data analysis, was used to extrapolate relevant text to describe the psychological interventions. Another member of the team reviewed the transcripts to confirm or dispute the coding attributed by the first reviewer. All transcripts included for analysis were reviewed by two team members. Subsequently, the two reviewers worked collectively to describe the core meaning of the included content while also selecting specific interviewees’ quotes to represent the interviewees’ thoughts as needed.

## Results

### Results of the literature review

**Fig. 1:**
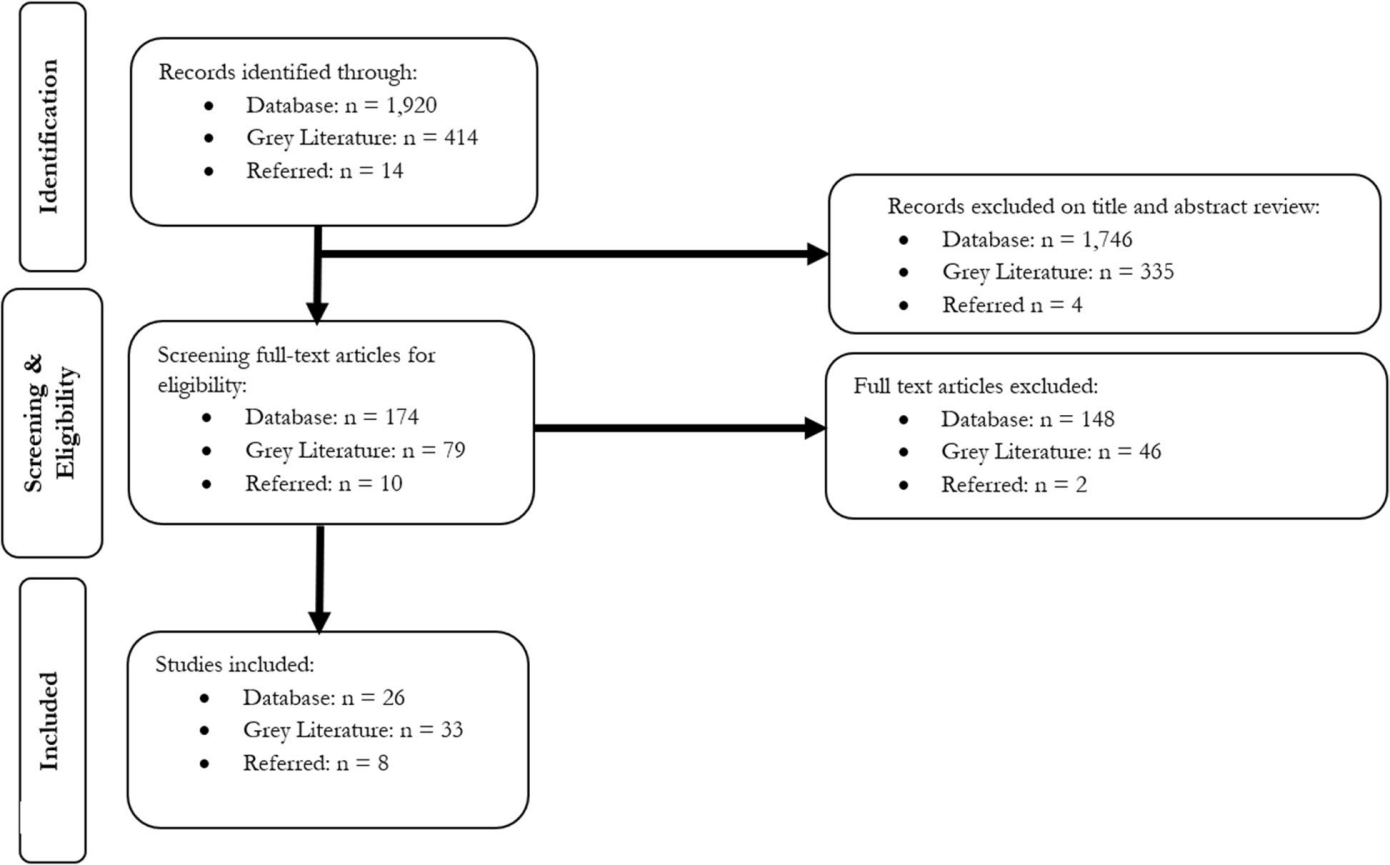
PRISMA flow diagram

**Fig. 2:**
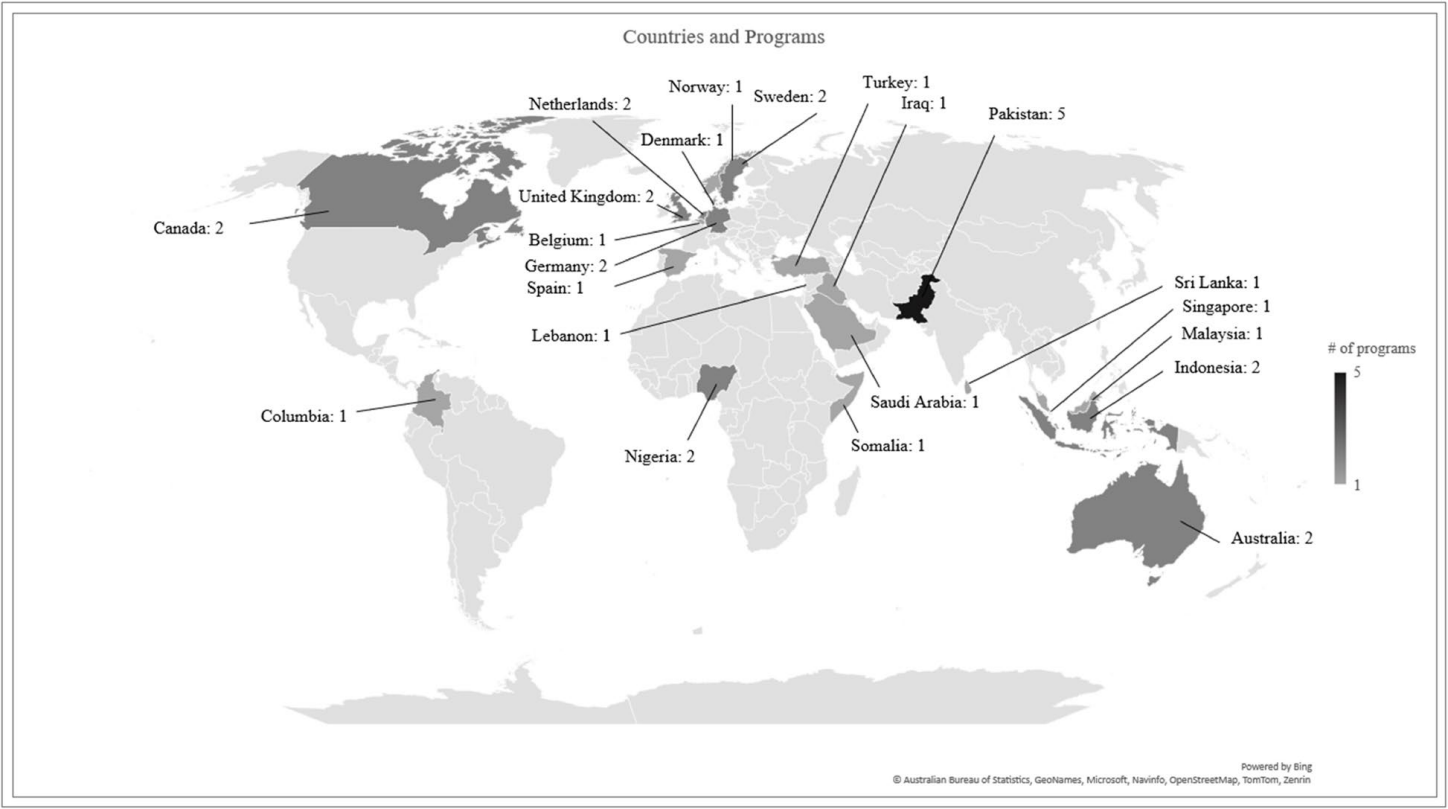
Number of programs by country

We screened a total of 2,348 articles and reports of which 1,920 were scientific articles, 414 were grey literature reports, and 14 represented additional articles/reports suggested by the experts (see Fig. 1). After reviewing the titles and abstracts, 263 articles were included for full-text review, of which 174 were scientific articles, 79 were gray literature reports, and 10 were articles referred by experts. Finally, after the full-text review was completed, we retrieved 67 articles and reports meeting our inclusion criteria (a full reference list of these articles can be found in Appendix IV). These 67 articles described 34 programs (some programs were discussed in more than one article) for which there was information on the use of one or more psychological interventions as part of tertiary programs. These 34 programs were located across 22 countries (Fig. 2), of which the majority (*n* = 12) were situated in Europe, 10 in South and Southeast Asia, four in the Middle East, three in Sub-Saharan Africa, three in North and South America, and two in Australia.^2^ Below we summarize our main findings for each program with a full list presented in Table 7 (see Appendix IV). Specifically, we summarize our findings based on the following criteria: 1) information on the specific type of psychological treatments used in the tertiary programs, and 2) key modalities surrounding the setting and delivery of such psychological interventions that can have important implications on the quality of care delivered. With regards to the latter, this includes: the type of personnel involved in delivering psychological treatment; the duration of the psychological treatment; whether care was delivered individually or in a group setting; whether participation was voluntary; whether beneficiaries’ risk (ideological commitment or risk to engage in violence) was considered when selecting individuals to receive these interventions; and the setting of these programs.

### Specific psychological treatments

Out of the 34 programs that we identified as including a psychological intervention, the level of detail in the descriptions of such interventions varied significantly (see Appendix II Table 1 for a greater description of the psychological treatments identified). We found very detailed examinations of the effects of the psychological interventions, as is the case in Muluk et al.’s (2020) study of Indonesia’s deradicalization program (Indonesia-P) and Peracha et al.’s (2022) assessment of Pakistan’s Sabaoon Center. In the case of Pakistan’s Sabaoon, the literature provided extensive details about the structure of the psychological intervention. Specifically, Sabaoon was described as utilizing the integrative thinking method to promote greater cognitive complexity and perspective-taking among beneficiaries.^3^ However, in other cases, the literature only briefly mentioned the psychological treatments included in the program.

Out of the 34 programs identified in the literature, 10 were described in sufficient detail to extract information about the type of psychological intervention used as part of the program. Such interventions included: emotional expression and cognitive flexibility skills training; aggression replacement therapy; functional family therapy; cognitive behavioral therapy; systems therapy; motivational interviewing, expressive therapy, and the house of healing method. For 10 additional programs we retrieved literature vaguely mentioning the use of informed approaches to therapy without further details. Such approaches included: mindfulness-based and trauma-informed approaches, as well as the use of counseling/therapy. Table 2 summarizes information on the programs that use these interventions and informed approaches. For a brief description of these approaches, refer to Table 1 in the Appendix II section of this manuscript. Literature on the remaining 14 programs only mentioned the use of a psychological approach without providing any further details.

**Table 2:** Therapies and informed-approaches

Country	Program	Specific Therapy	Informed Approaches
**South and Southeast Asia**			
Indonesia	YPP	-	-Trauma-informed approach
Indonesia	Indonesia-P	-Emotional expression skills -Cognitive flexibility skills	
Pakistan	Sabaoon	-Integrative thinking method	
Sri Lanka	6 + 1	-Expressive therapy	-Mindfulness-based approach
**Middle East**			
Saudi Arabia	PRAC	-Expressive therapy	
Iraq	IRPGS	-	-Trauma-informed approach
Lebanon	Rescue Me	-Expressive therapy -Aggression replacement therapy -Functional Family Therapy -House of healing method	
**Sub Saharan Africa**			
Nigeria	OSC	-Psychotherapy -Expressive therapy	
Nigeria	YRI	-	-Trauma-informed approach
Somalia	The National Programme	-	-Trauma-informed approach
**Europe**			
Belgium	The Belgium Model	-	-Trauma-informed approach
Denmark	Aarhus Program	-	-Trauma-informed approach
Sweden	Entré	-Cognitive-behavioral therapy	
Netherlands	Forsa Program	-	-Trauma-informed approach
Netherlands	DP	-Systems therapy -Cognitive-behavioral therapy	-Trauma-informed approach
Norway	NP	-Cognitive-behavioral therapy	
United Kingdom	HII^a^	-	-Mindfulness-based approach
**North America**			
Canada	Evolve Program	-	-Trauma-informed approach
Canada	The Quebec Model	-	-Trauma-informed approach
**Australia**			
Australia	PRISM	-Motivational interviewing -Cognitive-behavioral therapy	

^a^HII is not a program in the way that most others are on this list. Rather, it is an intervention offered to offenders, and can, for instance, be offered to offenders that are part of the Desistance and Disengagement Programme (DDP)

### Type of personnel involved in the interventions

Across the 34 programs identified in the literature, information on the key personnel involved in delivering the tertiary programs varied significantly across the literature reviewed. In some cases, reference to this information was vague, not mentioned, or difficult to discern. The personnel listed in the studies range from security actors, civil society mentors, former extremists, religious clerics, psychiatrists, psychologists, and social workers, among other mental health professionals, with the literature often listing more than one type of professional per program.^4^ Moreover, of the 24 programs for which we could find a description of the type of personnel involved (see Table 7 in Appendix IV), 15 listed mental health professionals (such as psychologists, therapists, counselors, psychiatrists, etc.) as having some form of engagement in the delivery of the psychological intervention.^5^ However, we could only confidently identify 13 programs for which the article made clear that the mental health providers, including psychologists, therapists, trained social workers, and/or general healthcare providers were directly involved in delivering therapies to the extremists. These 13 programs were based in Malaysia (Tafaquhh Fiddin Program (TFP)), Singapore (Singapore Program (SP)), Pakistan (Sabaoon, Rastoon, Mishal), Sri Lanka (6 + 1), Saudi Arabia (PRAC), Lebanon (RM), Somalia (National Programme), Sweden (Entré), Canada (Quebec), Columbia (Reincorporation Program (RP)), and Australia (PRISM).

Most programs included more than one type of practitioner.^6^ For instance, Australia’s PRISM program was delivered by a team of psychologists partnered with a religious support officer, a services and programs officer, and allied health professionals, among other figures not all of whom are involved in directly delivering psychological services (Cherney & Belton, 2020). In other cases, mental health professionals were recruited to train staff who would then provide psychological support to the extremists. For example, Sri Lanka’s rehabilitation centers brought in “external clinical psychologists and mental health workers to train staff on how to counsel beneficiaries” (Webber et al., 2018).

### Duration of the interventions

Most of the articles did not describe the duration of the program and/or information about the frequency and/or duration of the psychological interventions. Of the 34 programs identified, only 11 provided information about duration. However, it was sometimes unclear whether the duration indicated refers to the period beneficiaries spend receiving psychosocial support, or whether this also included other activities such as religious counseling and vocational training, among other services provided as part of the program. Accordingly, it was difficult to ascertain whether the duration specified is directly related to the psychological intervention, or if it was more broadly related to the program overall. This is important to note because the durations/frequencies indicated across the 11 programs range from a few days to years. The 11 programs identified included: Indonesia’s program (Indonesia-P) – ranging from two days to three months; Malaysia’s TFP – ranging from two to three years; Pakistan’s Sabaoon, Rastoon, and Mishal Centers – ranging from six months to three years; Sri Lanka’s 6 + 1 program – lasting roughly two years; Saudi Arabia’s PRAC – lasting twelve weeks; Iraq’s IRPGS – ranging from four to six weeks; Lebanon’s RM – occurring weekly except for holidays; Nigeria’s OSC – ranging from 26 to 52 weeks; Netherlands’ Forsa program – lasting up to one or two years.

### Group vs. individual therapy

Among the studies reviewed, information on session type was reported for 10 of the 34 programs (i.e., whether the psychosocial treatment is being delivered in either individual or group sessions). Among the 10 programs, 6 provided only individual counseling: Malaysia’s TFP; Pakistan’s Mishal; Sweden’s Entré; Sweden’s Exit Program (Exit-Sweden); Netherland’s Forsa; and Canada’s Quebec Model (Quebec). The remaining 4 programs provided both individual and group sessions: Indonesia-P; Saudi Arabia’s PRAC; Iraq’s IRPGS; and Lebanon’s RM. Notably, Saudi Arabia’s program initially provided one-on-one counseling, but beneficiaries could later participate in group therapy (Boucek, 2008). Furthermore, Iraq’s program consisted mainly of group sessions and only included individual sessions “as needed” (Speckhard, 2011), without specifying what would determine the need for individualized approaches.

### Program setting: government vs non-state programs

Most of the programs identified in our review (i.e., 28 out of the 34) were run by the government, at times in partnership with NGOs and other civil society organizations.^7^ Only five programs were primarily, or exclusively, run by non-state actors. These programs included Lebanon’s RM, Indonesia’s YPP, Germany’s Exit-Deutschland, Sweden’s Exit-Sweden, and Canada’s Evolve (see Appendix III Table 3). Based on information available in the articles reviewed, it was not possible to determine whether the remaining program, Netherland’s Forsa program, was run by the government or non-state actors.

### Program setting: custody-based programs

Only three of the tertiary prevention programs captured in this review were reported to be operating utside of custody/detention/prison settings, often preparing beneficiaries for reintegration into the community.^8^ These programs included Sweden’s Exit-Sweden, Canada’s Quebec Model, and Columbia’s RP (see Appendix III Table 4). The majority (24 out of the 34 programs) of the interventions identified were intended for extremists in custody.^9^ The articles that discussed the remaining seven programs – which were based in Indonesia (YPP), Turkey (Disengagement and Deradicalization Pilot Program (DDPP), Nigeria (YRI), Denmark (Aarhus), the Netherlands (Forsa), Canada (Evolve), and Australia (Intervention 01) – did not indicate whether the programs described were custody based.

Moreover, among the custody-based programs identified, only five (Sri Lanka’s 6 + 1, Saudi Arabia’s PRAC, Netherland’s DP, Malaysia’s TFP, and Somalia’s National Programme) were clearly identified in the literature as providing aftercare programs to monitor and assist with the reintegration process post-release from custody. For example, the literature described Saudi Arabia’s PRAC program as one of the most developed aftercare programs, where, as part of a halfway house, participants received art therapy, religious lectures, and additional social or vocational support to aid in their reintegration from prison (Speckhard, 2011).

### Participation: voluntary or mandatory

In the articles we reviewed, there was sufficient information to determine whether participation in the intervention was mandatory or not for just 19 of the programs. Among the 19 programs, 13 were voluntary. These programs were based in Western, Middle Eastern, African, and South American nations (see Table 5 in Appendix III). The remaining 6 programs required mandatory participation and were based in Belgium (BM), Norway (NP), the United Kingdom (DDP), Saudi Arabia (PRAC), Malaysia (TFP), and Singapore (SP).

Notably, for some voluntary programs, there were certain factors other than, or in addition to a willingness to change, that may incentivize an individual’s participation. For instance, although participation in Spain’s Framework Program was voluntary, those who successfully completed treatment received benefits such as comparable conditions as those for regular prisoners or being transferred to a prison close to their residence (RAN, 2019; Speckhard, 2011).^10^^, ^11 Furthermore, there were tertiary prevention programs that required participants to meet certain criteria prior to participation (Basit, 2015; Basra, 2022; Horgan & Braddock, 2010).^12^ For example, although the Somali program was voluntary, participants were required to disengage^13^ before participating, and the same was true for Pakistan’s Punjab program.

### Risk posed by the individual

Finally, we identified studies that mentioned risk (i.e., level of ideological commitment and support for a violent extremist cause) as an exclusion criterion for participation in the program (See Table 7 in Appendix IV). Of the 34 programs, eight were reported to exclusively provide services to low-risk individuals or, conversely, refuse to provide services to high-risk individuals. However, across these eight programs, different criteria qualified someone as low or high-risk.^14^ For example, Exit Deutschland and the Somali National Programme required individuals to disengage voluntarily prior to entering the program (Exit Deutschland, 2014; Khalil et al., 2019a). In other cases, the literature specified that the program only works with low-risk individuals but did not describe who qualified as low-risk. Operation Safe Corridor in Nigeria, for instance, was described as a program for “repentant, low-risk, male Boko Haram combatants” (Ugwueze et al., 2022, p. 1236). Additionally, of the 34 programs, six mentioned that they work with individuals regardless of risk. The Sabaoon Center in Pakistan, for example, explicitly provided services to both low- and high-risk extremist youth. Finally, 20 programs did not clearly specify whether risk level was a barrier to receiving services.

### Interview results

#### Psychological interventions mentioned by the interviewees

Below we provide information on the type of psychological interventions reported by the practitioners we interviewed. It is worth noting that these results do not reflect the success of the interventions, as we could not find literature that provides sufficient evidence on the efficacy of different psychological interventions used as part of tertiary programs. Instead, these results reflect what are considered as promising practices by the interviewees.^15^ The psychological interventions most frequently mentioned by the interviewees were: cognitive behavioral therapy (CBT) (*n* = 12), emotional regulation/distress tolerance techniques (*n* = 7), trauma informed care and trauma therapy (*n* = 6), and dialectical behavioral therapy (DBT) (*n* = 6). One interviewee working in Canada described using behavioral therapy with the goal of changing “*the individual’s behavioral engagement with violent extremism.*” Interviewees also mentioned the importance of building the foundations for a healthy lifestyle, acquiring and strengthening positive social relationships, and enhancing the client’s awareness of the consequences of maladaptive behaviors.

However, a licensed mental health provider working in the US noted that “*… CBT can be really effective, but I don't think it focuses on the root of certain problems*” suggesting that dialectical behavioral therapy (DBT) could serve as a more in-depth approach to address the reasons behind a violent extremist behavior. The use of DBT was mentioned by five other interviewees. As another interviewee from the US said: “*I always prefer DBT because I think oftentimes the root of maladaptive behavior is distress, like having poor distress tolerance or instant gratification problems.*”

Six interviewees discussed the importance of addressing trauma, because it is a frequent underlying cause of maladaptive behaviors. As described by an interviewee from Canada: “*Grievances are crystallized around past traumatic events.*” More specifically, four interviewees talked about the use of eye movement desensitization and reprocessing (EMDR), and two interviewees talked about the importance of using trauma-informed care approaches. A licensed mental health provider in the US also stressed that not everyone who experiences trauma needs trauma therapy as “*the most frequent reaction to trauma is resilience.”* Four interviewees talked about using approaches that help their clients recognize their emotions and come up with appropriate responses, and the need to work on emotional self-regulation and distress tolerance. Indeed, an interviewee from the US stated, *“I find that their distress tolerance really interferes with the “extremist” ability to change those cognitions and later change those behaviors.”*

Mindfulness was mentioned by four interviewees as an approach that could work with some clients, depending on their cultural background. In addition, the importance of reflective and active listening was mentioned by four interviewees as useful strategies to build rapport. Other approaches mentioned by three or fewer interviewees include: moral reconation therapy (MRT), acceptance and commitment therapy (ACT), motivational interviewing, psychodynamic therapy, applied behavioral analysis therapy (ABA-by an interviewee working with autistic individuals), rational behavioral therapy (RBT), cultural formulation interviewing (CFI), healthy identity interventions (an interviewee in the UK) and expressive therapy (by an interviewee in Lebanon). For a brief description of these approaches refer to Table 1 in Appendix II of this manuscript.

#### Personnel and setting of the interventions

Interviewees identified psychiatrists, psychologists and social workers, behavioral analysts, and substance use disorder therapists as of primary importance for delivering the psychological interventions to beneficiaries. Mentors from civil society and religious organizations were also highlighted as useful personnel. In addition, some interviewees suggested the need to include therapists with expertise in autism, as autism is not uncommon within the population of violent extremists.

Regarding the structure of the interventions, interviewees said they met their clients wherever it was most appropriate and safe. Sometimes they met them in their office, as well as in parks, café’, or the clients’ home when conditions allow it. Virtual interventions have also become more frequent after the pandemic. Individual therapy was certainly the preferred approach, with interviewees reporting group settings as not beneficial for this population. That said, many interviewees suggested that group therapy and counseling can be effective for close friends and family members. Furthermore, interviewees noted that the frequency of the sessions depended on the severity of the situation, with an average 50 min session held once a week increasing to multiple sessions per week for the most difficult cases. Interviewees further mentioned that the number of sessions delivered was often dependent on insurance coverage, which posed a common challenge in the delivery of mental health services in the US. Such limitations pose difficulties in achieving expected therapeutic goals, particularly for these types of clients for whom trust building is time-consuming.

#### Additional considerations expressed by the interviewees

Most interviewees (*n* = 14) emphasized the importance of working with the individual as well as with the family members, social network, and overall environment where the person is being re-integrated (i.e., school staff, employer, health professionals interacting with the extremist) to reduce the probability of rejection from society with insurgence of new conflicts and grievances during the reintegration process.

## Discussion

This study combined a scoping literature review with interviews to identify what type of psychological interventions are most frequently used in tertiary prevention programs for violent extremists. While several approaches have been mentioned in the literature, most articles do not describe with sufficient detail the intervention being used. The literature on the “Indonesian program” comes the closest to providing sufficient information to ascertain the: relevant therapies used with extremists; personnel involved; duration of the intervention; personnel involved; and structure of the intervention (whether group or individual sessions).

The results from the interviews allowed us to supplement the scarcity of literature on the topic and identify promising interventions, such as CBT and DBT, used with this population. The therapists we interviewed emphasized the need to recognize how trauma may affect behaviors in these individuals and the importance of using trauma informed approaches on a regular basis and trauma therapy only when needed. Interestingly, while there was limited literature published on United States-based programs, we were able to find several clinicians with direct experience in delivering psychological interventions to this type of clients. Most were doing it through private organizations that often do not have the capacity to publish their experience and results. The challenge in describing what interventions are most useful for violent extremists relies on the fact that there is no standardized approach in the delivery of the psychological treatments. Every practitioner is tailoring the intervention based on the needs of the client, frequently mixing a multiplicity of approaches and techniques. In addition, it should be expected that programs vary because they reflect the specific context, available resources, and culture of the population being served. It is worth noting that just as the treatment components vary across programs, the professional backgrounds of the providers vary as well with inconsistencies in access to clinicians. There is a particular concern that programs that lack access to mental health practitioners may resort to having untrained and unlicensed personnel serve as substitute therapists. For instance, interviews conducted with mentors who worked as part of a tertiary intervention in a Nordic country found that one challenge was having limited access to psychologists. This situation can lead mentors to serve as substitute therapists to detainees without the training and support to do so (Orban, 2022).

Most notably, psychological interventions are not delivered in isolation but rather frequently paired with social, economic, and at times religious interventions. The absence of literature that explicitly examines the measured impact of the psychological portion of a tertiary prevention program renders it impossible to isolate the potential positive outcomes of such interventions from the other program components (e.g., vocational training). In short, if the outcomes of psychological interventions are not formally accessed or evaluated there is no way to know what works and what does not work. To move the needle towards evidence-based approaches in this field, it is essential that practitioners and researchers develop data collection mechanisms that allow for a transparent and independent evaluation of the effectiveness of these psychological interventions and come to an agreement, with input from clients, on how to define success. Furthermore, in absence of universally agreed-upon definitions of deradicalization and disengagement, it is challenging to understand the aims of the tertiary prevention programs and consequently align the aims of the psychological interventions with overall program goals. Moving forward, researchers, policymakers, and program managers must be more transparent and diligent in documenting, describing, and discussing therapeutic interventions. Future research should focus on providing clear and detailed descriptions of the implementation of such approaches to make replication and evaluation efforts possible and support those that are in the process of developing new disengagement, deradicalization and/or reintegration efforts for violent extremists.

## Conclusion

Our findings demonstrate that tertiary prevention programs across the world utilize psychological interventions to facilitate the disengagement, deradicalization, and reintegration of extremists. A large focus on such interventions is behavioral change meaning a change in the participation in extremist criminal activities as well as a more general embracement of a healthy life and healthy relationships. Through the literature and the interviews, we were able to identify over a dozen psychological approaches with promising practices such as the use of cognitive, dialectical behavioral therapies and trauma informed and focused approaches. However, in most cases, it is not clear what specifically constitutes these treatments, how they are implemented, by whom and most importantly what are the goals of such interventions and of the tertiary prevention programs overall. Details on their implementation are also lacking. Most programs are vaguely described as offering “psychological counseling or support”. Future research should focus on documenting the delivering of psychological interventions so to offer an opportunity for replication by those who are developing tertiary prevention programs.

## Supplementary Information

Supplementary Material 1.

## Data Availability

Interview data will be uploaded onto the National Archive of Criminal Justice Data once the grant is closed. In the meantime, data will be made available to the reader upon reasonable request.

## Footnotes

1 However, there is a lack of consensus around the conceptualization of tertiary prevention programs. For some, tertiary prevention programs are limited to persons “with demonstrated violent behavior” (Weine et al., 2016, pg. 9). For others, tertiary prevention programs not only include those who have demonstrated violent behavior, but also those that are associated with a violent extremist group whether or not this involves direct engagement in violence (Khalil et al., 2019b). In this scoping review, we rely on this latter conceptualization of tertiary prevention programs, because we believe its inclusivity better reflects the scope of the existing tertiary prevention programs across the world.

2 See Table 1 in Appendix II for a summary of the program breakdown by country.

3 Notably, Integrative Complexity is a commonly utilized validated psychometric measure, however, in the case of Pakistan’s Sabaoon Center it is described as both a method of psychological intervention and an outcome measurement, thus justifying the current study’s inclusion in the specific reference to a therapy.

4 see Table 6 in Appendix III section for more detailed breakdown of results.

5 These 15 programs are based in: Malaysia (TFP); Singapore (SP); Pakistan (Sabaoon, Rastoon, and Mishal); Sri Lanka (6 + 1); Saudi Arabia (PRAC); Iraq (Iraq Rehabilitation); Nigeria (OSC); Nigeria (YRI); Sweden (Entre); Netherlands (Forsa); Canada (Evolve Program, and Quebec Model); and Australia (PRISM).

6 see Table 6 in Appendix III section for more detailed breakdown of results.

7 See Table 6 in Appendix III section for more detailed breakdown of results.

8 See Table 6 in Appendix III section for more detailed breakdown of results. It is worth mentioning that though the literature included in this review indicates that some programs operate outside of custody/detention centers, it does not mean that they may not also provide services during detention.

9 It is worth noting, however, that though Germany’s EXIT-Deutschland and the United Kingdom’s Healthy Identity Intervention provide services for individuals in prison, these programs are also available to those outside of prison.

10 In Belgium’s case, participation is only mandatory to some as a condition of probation while others may volunteer (see, RAN, 2019).

11 Similarly though participation is voluntary as part of Iraq’s Rehabilitation Program, those who participate have the potential for accelerated release. Meanwhile, although Malaysia’s program was mandatory, it is likely participants were compliant due to the coercive nature of the intervention (e.g., participants were beaten if they resisted) (Speckhard, 2011).

12 One criterion was age as the Sabaoon and Rastoon Centers are only for youth participants while the Mishal Center is only for adults (Basit, 2015; Basra, 2022). Moreover, the Punjab program required participants to have disengaged prior to partaking in the program (Basit, 2015). The single South American program based in Columbia, the Reincorporation Program, is voluntary, though participants must meet conditions such as, but may not be limited to, expressing a wish to demobilize, surrendering weapons, and providing a private report on their participation in illegal acts (Horgan and Braddock, 2010). Once in the program, participants have to continue to check in with one of the many government reference centers.

13 It is important to note, however, disengaged al-Shabaab members who do not choose the rehabilitation program risk could to prison instead, which limits their options.

14 For the Iraqi Program, rather than risk being used as an exclusion criteria, it is used as a sorting criteria. That is, beneficiaries presenting with different risk levels (i.e., moderate and high risk) are sorted into programs that are tailored to their risk. For instance, those that are “high risk” (i.e., those dedicated to militant jihadi ideologies or were engaged in sectarian violence, or engaged in violence as economic opportunists) not only received psychological treatment of their traumas, as well as religious counseling intended to challenge militant jihadi ideologies. Meanwhile, those considered moderate were provided with a shorter program that addressed Islamic values and provided psychological values (Spechard, 2011).

15 It is worth noting that psychological interventions are often just one component of tertiary prevention programs. other components can include religious interventions, vocational training, social support etc.
