# Supplementary material for: The use of psychological interventions in tertiary prevention programs for individuals engaged in violent extremism: a scoping review and interviews
Source: Health Justice. 2025 Mar 22;13:17. doi: 10.1186/s40352-025-00324-w (PMC11929306; doi:10.1186/s40352-025-00324-w)
Supplement: Supplementary file 1 — Supplementary Material 1. [file 40352_2025_324_MOESM1_ESM.docx]

**APPENDIX I**

**Grey Literature Sources**

Grey literature was searched through Google’s search function at the URL level using the following two search strings (each site was searches using the two strings):

Search String 1: site: rand.org filetype:pdf deradical* OR disengag* OR reintegrat* OR demobili* OR "countering violent extremism"

Search String 2: site: rand.org filetype:pdf deradical* OR disengag* OR reintegrat* OR demobili* OR "countering violent extremism" AND "Therapy"

1. Australian Strategic Policy Institute
2. Brookings Institution
3. Combating Terrorism Center at West Point
4. Carnegie Endowment for International Peace
5. The Center for Strategic and International Studies (CSIS)
6. Council on Foreign Relations (CFR)
7. Counter-Terrorism Committee of the United Nations (UNCTC)
8. Program on Extremism at George Washington University (GWUPOE)
9. German Institute of Radicalization and Deradicalization Studies (GIRDS)
10. Global Counterterrorism Forum (GCTF)
11. International Center for Counter-Terrorism (The Hague) (ICCT)
12. Institute for Strategic Dialogue (ISD)
13. International Institute for Counter-Terrorism (ICT)
14. International Peace Institute (IPI)
15. Leiden University
16. Organization for Security and Cooperation in Europe (OSCE)
17. RAN (Radicalization Awareness Network)
18. RAND Corporation
19. Resolve
20. The Soufan Group
21. National Consortium for the Study of Terrorism and Responses to Terrorism (START)
22. Terrorism Prevention Branch, United Nations Office on Drugs and Crime (TPB/UNODC)
23. United States Institute of Peace (USIP)
24. Wilson Center
25. Egmont Royal Institute for International Relations
26. International Center for Political Violence and Terrorism Research (ICPVTR) (within the S. Rajaratnam School of International Studies (RSIS))
27. The International Center for the Study of Radicalisation (ICSR)
28. Danish Institute for International Studies
29. Norwegian Institute of International Affairs (NUPI)
30. CNP-PREV
31. UNESCO-PREV CHAIRE
32. Moonshot
33. Life After Hate (EXIT USA)
34. EXIT-Deutschland
35. Hedayah
36. Addressing Violent Extremism and Radicalisation to Terrorism (AVERT)
37. C-REX Center for Research on Extremism
38. Office of Justice Programs
39. Royal United Services Institute RUSI
40. IMPACT EUROPE
41. COUNCIL OF EUROPE
42. UNESCO - UNESDOC
43. The Global Center on Cooperative Security
    1. [Global Center on Cooperative Security-Global Center on Cooperative Security](https://www.globalcenter.org/)
44. Defense Technical Information Center (DTIC)
45. European Union Institute for Security Studies
46. The International Centre for the Study of Radicalisation and Political Violence
47. Middle East Institute (MEI)
48. War on The Rocks
49. Violence Prevention Network
50. Institute for Security Studies (ISS)
51. TerRa (Terrorism and Radicalisation)
52. Combating Terrorism Center
53. Swedish National Council for Crime Prevention (Brottsförebyggande rådet, BRÅ)
54. National Institute of Justice (NIJ)
55. The Unity Initiative
56. US Director of National Intelligence (DNI)
57. US Department of Homeland Security (DHS)
58. US Government Accountability Office (GAO)
59. US National Counterterrorism Center (NCTC)
60. NCITE
61. The UN Center for Counter-Terrorism

**APPENDIX II**

**Table I: Description of Psychological Interventions**

| **Psychological Intervention** | **Description verbatim from sources** | **Source** | **Intervention discussed in article or interview** |
| --- | --- | --- | --- |
| Acceptance and Commitment Therapy (ACT) | “… empirically based psychological intervention that uses acceptance and mindfulness strategies, together with commitment and behavior change strategies, to increase psychological flexibility.” | https://contextualscience.org/act | Interview |
| Moral Reconation Therapy | “The term ‘moral’ refers to moral reasoning based on Kohlberg's levels of cognitive reasoning. The word ‘reconation’ comes from the psychological terms ‘conative’ and ‘conation,’ both of which refer to the process of making conscious decisions. MRT is a cognitive-behavioral treatment system that leads to enhanced moral reasoning, better decision making, and more appropriate behavior.” | https://www.moral-reconation-therapy.com/ | Interview |
| Psychodynamic Therapy | “The basic goals of psychodynamic psychotherapy are to:  1. understand elements of the patient’s unconscious that are affecting his/her conscious thoughts, feelings, and behavior  2. decide whether uncovering or supporting will help most at that moment  3. uncover unconscious material or support mental functioning in the way that will best help the patient.” | Cabaniss, D. L., Cherry, S., Douglas, C. J., & Scwartz, A. (2011). The treatment for a mind in motion. In *Psychodynamic Psychotherapy: A clinical Manual* (pp 3-7). Wiley-Blackwell. | Interview |
| Applied Behavior Analysis | “helps us to understand:   - How behavior works - How behavior is affected by the environment - How learning takes place   … applies our understanding of how behavior works to real situations. The goal is to increase behaviors that are helpful and decrease behaviors that are harmful or affect learning.” | https://www.autismspeaks.org/applied-behavior-analysis | Interview |
| Cultural Formulation Interview | “…to change the way clinicians conduct a diagnostic interview so that the perspective of the patient becomes at least as important as the signs and symptoms of disease identified by the clinician. The mission of the CFI is in fact to expand what counts as data in a clinical encounter, encouraging the clinician—and the patient, who is empowered to recount his or her experience more fully—to attend to the experience of illness and the life world.” | Aggarwal, N. K., & Lewis-Fernandez, R. (2020). An introduction to the Cultural Formulation Interview. *Focus: The Journal of Lifelong Learning in Psychiatry, 13*, 426-431. <https://doi.org/10.1176/appi.focus.18103> | Interview |
| Rational Behavioral Therapy | “… a comprehensive, culture-free, shortterm, cognitive-behavior therapy. It is comprehensive because it deals directly with all three groups of learned behaviors: the cognitive, the emotive, and the physical.” | Maultsby, M.C. (1982). Rational Behavior Therapy. In: Turner, S.M., Jones, R.T. (eds) Behavior Modification in Black Populations (pp. 151). Springer, Boston, MA. https://doi.org/10.1007/978-1-4684-4100-0_8 | Interview |
| Aggression Replacement Therapy | “Features three coordinated and integrated components:   - social skills training: teaches participants what to do, helping them replace antisocial behaviors with positive alternatives. - anger control: teaches participants what not to do, helping them respond to anger in a nonaggressive manner and rethink anger-provoking situations.   moral reasoning: helps raise participants’ level of fairness, justice, and concern for the needs and rights of others.” | https://aggressionreplacementtraining.com/ | Article |
| Functional Family Therapy | “… short-term, evidence-based family-based counseling service designed for 11-to-18-year-old youth who are at risk or have been referred for behavioral or emotional problems. FFT works with a young person’s entire family and extrafamilial influences to facilitate positive growth and development… a phasic program with steps, which build upon each other. These phases consist of: engagement, motivation, relational assessment, behavior change, and generalization.” | https://www.fftllc.com/fft | Article |
| Cognitive Behavioral Therapy | “…trained therapists help clients identify distressing thoughts and evaluate how realistic these thoughts are. As clients become aware of their thoughts and are able to evaluate them, they feel better. CBT therapists also work with clients on solving problems, learning new skills, and setting and achieving meaningful goals. Although initially therapists and clients work together in session, therapists also empower clients by teaching them to evaluate their thoughts and practice their new skills on their own, outside of therapy.” | https://cares.beckinstitute.org/about-cbt/ | Article |
| Systems Therapy | A type of psychotherapy that focuses on the relationships between individuals and the systems (e.g., families, organizations, or communities) in which they exist. | https://mantracare.org/therapy/therapy-types/systems-therapy | Article |
| Motivational Interviewing | “MI is a collaborative, goal-oriented style of communication with particular attention to the language of change. It is designed to strengthen personal motivation for and commitment to a specific goal by eliciting and exploring the person’s own reasons for change within an atmosphere of acceptance and compassion.” | Miller, W.R. & Rollnick, S. (2013) *Motivational Interviewing: Helping people to change* (3rd Ed, pp. 29). Guilford Press. | Article |
| Expressive Therapy | “… the telling of a story through an expressive modality such as art, music, movement, etc.” | Malchiodi, C. A. (2005). Expressive therapies: History, theory, and practice. In C.A. Malchiodi (Ed.) *Expressive Therapies* (pp. 10). Guilford Press. | Article |
| House of Healing Methods | “A 13-session social emotional learning curriculum created specifically for prisoner rehabilitation… a trauma informed, mindfulness based, cognitive behavioral curriculum designed to equip incarcerated individuals with greater self awareness while increasing their capacity to manage difficult emotions.” | https://lionheart.org/lionheart-programs/houses-of-healing | Article |
| Emotional expression skills training | “… administered as a practice of knowing, specifying, and describing emotion through writing. Three aspects assessed were emotion regulation, evaluating the solution, and communicating ideas.” | Muluk, H., Umam, A. N., & Milla, M. N. (2020). Insights from a deradicalization program in Indonesian prisons: The potential benefits of psychological intervention prior to ideological discussion. *Asian Journal of Social Psychology, 23*, p. 48. https://doi.org/10.1111/ajsp.1239 | Article |
| Cognitive flexibility skills training | “… administered as a means of practicing problem-solving through consideration of different alternatives. Three aspects assessed were problem identification, accessing alternative solution for a given problem, and choosing a realistic solution.” | Muluk, H., Umam, A. N., & Milla, M. N. (2020). Insights from a deradicalization program in Indonesian prisons: The potential benefits of psychological intervention prior to ideological discussion. *Asian Journal of Social Psychology, 23*, p. 48. https://doi.org/10.1111/ajsp.12392 | Article |

**APPENDIX III**

**Table 2: Program and Country Breakdown**

| **Region** | **Country** | **Program** |
| --- | --- | --- |
| **South and Southeast Asia: 10 programs** | | |
|  | Indonesia | Outreach Division of the Yayasan Prasasti Perdamaian (YPP) |
|  | Indonesia | Indonesia Program (Indonesia-P) |
|  | Malaysia | Tafaquhh Fiddin Program (TFP) |
|  | Singapore | Singapore Program (unclear program name) (SP) |
|  | Pakistan | Sabaoon Center (Sabaoon) |
|  | Pakistan | Rastoon Center (Rastoon) |
|  | Pakistan | Mishal Center (Mishal) |
|  | Pakistan | Militant Rehabilitation Programme in Punjab (Punjab) |
|  | Pakistan | Bajaur District (Bajaur) |
|  | Sri Lanka | 6+1 Program (6+1) |
| **Middle East: 4 Programs** | |  |
|  | Saudi Arabia | Prevention, Rehabilitation, and Postrelease Care (PRAC) |
|  | Iraq | Iraqi Rehabilitation Program under General Stone (IRPGS) |
|  | Lebanon | Rescue Me |
|  | Turkey | The Disengagement and Deradicalization Pilot Program (DDPP) |
| **Sub Saharan Africa: 3 Programs** | |  |
|  | Nigeria | Operation Safe Corridor (OSC) |
|  | Nigeria | Yellow Ribbon Initiative (YRI) |
|  | Somalia | National Programme for the Treatment and  Handling of Disengaged Combatants in Somalia (National Programme |
| **Europe: 12 Programs** | |  |
|  | Belgium | The Belgium Model |
|  | Denmark | Aarhus Program (Aarhus) |
|  | Sweden | Entré Program (Entré) |
|  | Sweden | Exit-Sweden |
|  | Germany | German Program (unclear program name) |
|  | Germany | Exit Deutschland (Exit-D) |
|  | Netherlands | Forsa Program (Forsa) |
|  | Netherlands | The Dutch Program (unclear program name) (DP) |
|  | Norway | The Norwegian Program (NP) |
|  | Spain | Framework Program for Intervention in Violent Radicalization with Islamist Inmates (Framework Program) |
|  | United Kingdom | Desistance and Disengagement Program (DDP) |
|  | United Kingdom | Healthy Identity Intervention (HII) |
| **North America: 2 Programs** | |  |
|  | Canada | Evolve Program (Evolve) |
|  | Canada | The Quebec Model (Quebec) |
| **South America: 1 Program** | |  |
|  | Columbia | Reincorporation Program (RP) |
| **Australia: 2 Programs** | |  |
|  | Australia | PRISM |
|  | Australia | Intervention 01 (Intervene-01) |

Note. Acronyms used are developed by authors for ease of reporting

**Table 3: Programs Run by Non-State Actors**

| **Non-State Program** | **Country** | **Program** |
| --- | --- | --- |
| Yes | Indonesia | Outreach Division of the Yayasan Prasasti Perdamaian |
| Yes | Lebanon | Rescue Me |
| Yes | Sweden | Exit-Sweden |
| Yes | Germany | Exit Deutschland |
| **NPA** | The Netherlands | Forsa Program |
| Yes | Canada | Evolve Program |

Note. NPA: not publicly available

**Table 4: Non-Custody Based Programs**

| **Country** | **Program** |
| --- | --- |
| Sweden | Exit-Sweden |
| Canada | The Quebec Program |
| Columbia | Reincorporation Program |

Note. NPA: not publicly available

**Table 5: Voluntary and Mandatory Participation**

| Participation | No. of programs | Country | Name of programs |
| --- | --- | --- | --- |
| **Western Programs** |  |  |  |
| Mandatory | 3 | Belgium  Norway  United Kingdom | Belgium Model  Norwegian Program,  Desistance and Disengagement Program |
| Voluntary | 8 | Denmark  Sweden  Germany  Netherlands  Spain  United Kingdom  Canada  Australia | Aarhus Program  Exit-Sweden  Exit Deutschland  Forsa Program  Framework Program  Healthy Identity Intervention  Evolve Program  PRISM |
| Unknown | 5 | Sweden  Germany  Netherlands  Canada  Australia | Entrée Program  German Program  Dutch Program  Quebec Model  Intervention 01 |
| **Middle East and Africa Programs** | |  |  |
| Mandatory | 1 | Saudi Arabia | PRAC program |
| Voluntary | 4 | Iraq  Lebanon  Somalia  Nigeria | Rehabilitation Program  Rescue Me  The National Programme  Operation Safe Corridor |
| Unknown | 2 | Turkey  Nigeria | Disengagement and Deradicalization Pilot Program  Yellow Ribbon Initiative |
| **South and Southeast Asia Programs** | |  |  |
| Mandatory | 2 | Malaysia  Singapore | Tafaquhh Fiddin program  Singapore Program |
| Unknown | 8 | Pakistan  Pakistan  Pakistan  Pakistan  Pakistan  Sri Lanka  Indonesia  Indonesia | Sabaoon Center  Rastoon Center  Mishal Center  Militant Rehabilitation Programme in Punjab  Bajaur District  6+1 program  Indonesia program  Outreach Division of the Yayasan Prasasti Perdamaian |
| **South America Program** | |  |  |
| Voluntary | 1 | Columbia | Reincorporation program |

***Intended Program Outcomes: Deradicalization, Disengagement, and Reintegration***

There are notable geographic differences in the intended outcomes of the programs identified in this review. Specifically, the vast majority of interventions in Africa, South and Southeast Asia, and the Middle East identify deradicalization and reintegration as key goals (see Table 5 in Appendix II).^^[[1]](#footnote-1)^^ Conversely, most programs spanning Europe, Oceania, and North America attempt to facilitate disengagement and reintegration but not deradicalization.^^[[2]](#footnote-2)^^ There are some programs, however, that are exceptions to these trends. For example, three interventions were described as pursuing reintegration as the sole intended outcome: the Somalian SP, the Danish RP, and the Columbian RP. For each of these programs, reintegration may be the only intended outcome because participation is limited to those who have disengaged prior to entering the program.

Among Western interventions that do not list deradicalization as an intended outcome, the Evolve Program and the Belgian model help individuals deradicalize on a case-by-case basis. In the Evolve program, for instance, deradicalization is only an intended outcome for participants that are willing to deradicalize. In contrast to other Western interventions, the UK’s DDP and Exit-Deutschland are the only two for which reporting indicates that deradicalization is a program outcome for all individuals, in addition to reintegration and disengagement. Exit-Deutschland (2014) specified that “the essential core of our philosophy is, that the critical reassessment of the person’s past and the dismantling of the radical ideology are absolutely necessary to leave a radical milieu” (p. 5). Similarly, the DDP aims to help individuals adopt more mainstream religious beliefs and an identity that is more accepting of others.

**Table 6: Broad Intended Outcome(s) of Programs**

| Intended Outcome(s) | No. of programs | Country | Name of programs |
| --- | --- | --- | --- |
| **Region: South and Southeast Asia and the Middle East** | | | |
| Deradicalization and Reintegration | 7 | Malaysia  Singapore  Pakistan  Pakistan  Sri Lanka  Lebanon  Saudi Arabia | Tafaquhh Fiddin Program  Singapore Program  Sabaoon Center  Rastoon Center  6+1 Program  Rescue Me Program  PRAC Program |
| Disengagement and Reintegration | 2 | Iraq  Indonesia | Rehabilitation Program  Indonesian program |
| Disengagement, Deradicalization, and Reintegration | 2 | Turkey  Indonesia | Disengagement and Deradicalization Pilot Program  Outreach Division of the Yayasan Prasasti Perdamaian |
| Deradicalization | 1 | Pakistan | Mishal Center |
| Unknown | 2 | Pakistan  Pakistan | Militant Rehabilitation Programme in Punjab  Bajaur District |
| **Region: Africa** | |  |  |
| Deradicalization and Reintegration | 2 | Nigeria  Nigeria | Operation Safe Corridor  Yellow Ribbon Initiative |
| Reintegration | 1 | Somalia | The National Programme |
| **Region: Europe, Oceania, and North and South America** | | |  |
| Disengagement and Reintegration | 10 | Belgium  Sweden  Sweden  Germany  Netherlands  Netherlands  Norway  Spain  Canada  Canada  Australia | Belgium Model  Exit Program  Entré Program  German Program  Dutch Program  Forsa Program  Norwegian Program  Framework Program  Evolve Program  Quebec Model  PRISM |
| Disengagement, Deradicalization, and Reintegration | 2 | Germany  United Kingdom | Exit Deutschland  Desistance and Disengagement Program |
| Reintegration | 2 | Denmark  Columbia | Aarhus Program  Reincorporation Program |
| Disengagement | 1 | United Kingdom | Healthy Identity Intervention |
| Unknown | 1 | Australia | Intervention 1 |

**APPENDIX IV: Full Results Table**

**Table 7: Tertiary Programs Identified in Review**

| Region | Program  Name; Country | Public Program (yes/no) | Custody-Based (yes/no) | Intended Outcomes | Participation | | Psychological Component  (specific/vague) | Social Component | Key Personnel | References |
| --- | --- | --- | --- | --- | --- | --- | --- | --- | --- | --- |
| Southeast Asia | | | | | | | | | | |
|  | Outreach Division of the Yayasan Prasasti Perdamaian;  **Indonesia** | No  Partners with Indonesian government  (see below) | NPA | Reintegration  Disengagement | NPA | **Vague-informed approach:**  -Trauma-informed approach | | Vocational training | NPA | Schenk et al., 2021 |
|  | Indonesian Program;  **Indonesia** | Yes  Partners with multiple civil society organizations | Yes | Reintegration  Deradicalization  Disengagement | NPA | **Specific:**  -Emotional expression skills  -Cognitive flexibility skills | | -Economic assistance  -Family assistance  -Religious counseling/ education/ mentoring | -Security personnel (police interrogators)  -Former extremists  -Religious clerics | Milla et al., 2020  Muluk, et al., 2020  Ezzarqui, 2010  Gunaratna, 2021  Rabasa et al., 2010 |
|  | Tafaquhh Fiddin Program;    **Malaysia** | Yes | Yes  Offers aftercare | Reintegration  Deradicalization | Mandatory | Vague | | -Social counseling  -Moral education  -Vocational training  -Economic assistance  - Religious counseling/ education/ mentoring | -Clerics  -Psychologists  -Social workers | Speckhard, 2011  El-Said, 2012  Bryans, 2016  Schenk et al., 2021  Gunaratna, 2021 |
|  | Singapore Program (unclear program name);  **Singapore** | Yes | Yes | Deradicalization  Reintegration | Mandatory | Vague | | -Vocational training  -Family assistance  -Religious counseling/ education/ mentoring | -Psychologists  -Religious scholars  -Security officers | Rabasa et al., 2010  Mabborang, 2019  Jayakumar, 2020  Fink & Hearne, 2008  Schenk et al., 2021  Alsubaie, 2017 |
| South Asia | | | | | | | | | | |
|  | Swat De-Radicalization Model  Sabaoon Center  **Pakistan** | Public-Private Partnership | Yes | Reintegration  Deradicalization | NPA  Only for youth | **Specific:**  -Integrative thinking method | | - Educational and vocational support  -Religious counseling/ education/ mentoring | -Psychologists  -Social Workers  -Doctors  -Religious Clerics | Peracha et al., 2016  Basit, 2015  ICPVTR & RSIS, 2010a  Khan, 2015 |
|  | Swat De-Radicalization Model  Rastoon Center  **Pakistan** | Public-Private Partnership | Yes | Reintegration  Deradicalization | NPA  Only for youth | Vague | | - Educational and vocational support  -Religious counseling/ education/ mentoring | -Psychologists  -Social Workers  -Doctors  -Religious Clerics | Peracha et al., 2016  Basit, 2015  ICPVTR & RSIS, 2010a  Khan, 2015 |
|  | Swat De-Radicalization Model  Mishal Center;  **Pakistan** | Public-Private Partnership | Yes | Deradicalization | NPA  Only for adults | Vague | | -Family assistance  -Vocational training  -Economic assistance  -Religious counseling/ education/ mentoring | -Psychologists | Basra, 2022  Kapur, 2020 |
|  | Militant Rehabilitation Programme in Punjab;  **Pakistan** | Yes | Yes | NPA | Must have disengaged prior | Vague | | -Vocational training  -Religious counseling/ education/ mentoring | NPA | Basit, 2015 |
|  | Bajaur District;  **Pakistan** | Yes | Yes | NPA | NPA | Vague | | - Educational and vocational support  -Religious counseling/ education/ mentoring | NPA | Basit, 2015 |
|  | 6+1 Program;  **Sri Lanka** | Public-private partnership | Yes  Offers aftercare | Reintegration  Deradicalization | NPA | **Specific:**  -Mindfulness-based approach  -Expressive therapy | | -Educational and vocational support  -Vocational Support  -Social reintegration training  -Cultural rehabilitation  -Leisure activities  -Religious rehabilitation | -Clinical psychologist  -Mental health workers | Hassan et al., 2021  ICPVTR & RSIS, 2010b  Basra, 2022  Bryans, 2016  Schenk et al., 2021  Webber et al., 2018  Seifert, 2010  Hettiarachchi, 2019  Hettiarachchi, 2018  Hettiarachchi, 2013 |
| Middle East | | | | | | | | | | |
|  | Prevention, Rehabilitation, and Postrelease Care;  **Saudi Arabia** | Yes | Yes  Offers aftercare | Reintegration  Deradicalization | Mandatory | **Specific:**  -Expressive therapy | | -Financial assistance to families  -Economic empowerment  -Healthcare  -Housing Assistance  -Arranging marriage  -Religious counseling/ education/ mentoring | -Within the Psychological and Social Subcommittee:  -Psychologists  -Psychiatrists  -Researchers | Basra, 2022  El-Said, 2012  Rabasa et al., 2010  Speckhard, 2011  Davis & Cragin, 2009  Boucek, 2008a  Casptack, 2015  Hoeft, 2015  Fink & Hearne, 2008  Horgan & Braddock, 2009  UNODC, 2021  Alsubaie, 2017  Horgan & Braddock, 2010  Ezzarqui, 2010  Stern, 2010  Boucek, 2008b  Hill, 2016 |
|  | Iraqi Rehabilitation Program under General Stone;  **Iraq** | Yes  Funded by the U.S. Department of Defense | Yes | Reintegration  Deradicalization  Disengagement | Voluntary  Potential for accelerated release  High-risk individuals not eligible | **Vague-informed approach:**  -Trauma-informed approach | | -Educational and vocational support  -Literacy training  -Religious counseling/ education/ mentoring | -Psychologists  -Clerics | Speckhard, 2011  Rabasa et al., 2010 |
|  | Rescue me;  **Lebanon** | No | Yes | Reintegration  Deradicalization | Voluntary | **Specific:**  -Expressive therapy  -Aggression replacement therapy  -Functional Family Therapy  -House of healing method | | Outsources social support | Social workers | Anderlini & Holmes, 2019 |
|  | The Disengagement and Deradicalization Pilot Program;  **Turkey** | Yes | NPA | Reintegration  Deradicalization    Disengagement | NPA | Vague | | -Vocational training  -Employment and housing assistance  -Healthcare | NPA | Hassan et al., 2021 |
| Sub Saharan Africa | | | | | | | | | | |
|  | Operation Safe Corridor;  **Nigeria** | Yes | Yes | Reintegration  Deradicalization | Voluntary | **Specific:**  -Expressive therapy | | - Educational and vocational support  -Religious counseling/ education/ mentoring | -Psychologists  -Social workers  -Educators  -Artisans  -Religious leaders  -Deradicalization specialists | UNODC, 2021  Ugwueze et al., 2022  Onapajo & Ozden, 2020  International Crisis Group, 2021  Barkindo & Bryans, 2016 |
|  | Yellow Ribbon Initiative;  **Nigeria** | Public-Private  partnership between NEEM foundation and Borno State | NPA | Reintegration  Deradicalization | NPA | **Vague-informed approach:**  -Trauma-informed approach | | -Educational and vocational support  -Leisure activities  -Food and healthcare  -Religious counseling/ education/ mentoring | -Psychologists | Nemr et al., 2018  Anderlini & Holmes, 2019  UNODC, 2021 |
|  | The National Programme;  **Somalia** | Yes | Yes | Reintegration | Voluntary  Must have disengaged prior | **Vague-informed approach:**  -Trauma-informed approach | | -Educational and vocational support  -Family reconnection  -Civic, political  -Religious counseling/ education/ mentoring | -Social workers  -General healthcare  providers | Hassan et al., 2021  Khalil et al., 2019  Zeuthen, 2021  UNODC, 2021 |
| Europe | | | | | | | | | | |
|  | The Belgium Model;  **Belgium** | Yes  Run by the Flemish Government and CAPREV | Yes | Reintegration  Disengagement | Mandatory for some | **Vague-informed approach:**  -Trauma-informed approach | | - Educational and vocational support  -Relational support  -Religious support available | Social Workers | Renard, 2020  Radicalisation Awareness Network, 2019 |
|  | Aarhus Program;  **Denmark** | Yes  Collaboration between police, social authorities, and Danish Prison and Probation Services | NPA | Reintegration | Voluntary  Must have disengaged prior | **Vague-informed approach**  -Trauma-informed approach | | -Free medical care  -Family support and housing assistance  -Coaching in social and vocational skills  -Religious counseling/ education/ mentoring | -Trained mentors  -Police  -Probation officers | Rosand, 2020  Christensen & Bjorgo, 2018 |
|  | Entré Program;  **Sweden** | Yes  Swedish Prison and Probation Services | Yes | Reintegration  Disengagement | NPA | **Specific:**  -Cognitive-behavioral therapy | | - Assistance with housing and employment | Licensed therapists, without specialized knowledge of extremist ideologies | Carlsson, 2021 |
|  | Exit;  **Sweden** | No | No | Reintegration  Disengagement | Voluntary | Vague | | - Educational and vocational support | -Social workers  -Former extremists | Fransen, 2021  Christensen & Bjorgo, 2018 |
|  | German Program (unclear program name)  **Germany** | Yes | Yes | Reintegration  Disengagement | NPA | Vague | | - Educational and vocational support  -Coaching in social skills  -Work activities  -Recreational groups | NPA | Said, 2020 |
|  | Exit Deutschland;  **Germany** | No | Yes and no | Reintegration  Deradicalization  Disengagement | Voluntary | Vague | | -Social Restructuring  -Educational and vocational support | Case managers | EXIT Deutschland, 2014 |
|  | Forsa Program;  **Netherlands** | NPA  Subsidized by Dutch Government | NPA | Reintegration  Disengagement | Voluntary | **Vague-informed approach**  -Trauma-informed approach | | - Educational and vocational support -Assistance in rebuilding social network  -Religious support | -Psychologists  -Systemic therapists  -Counselors  -Theologians | Rosand, 2020  National Coordinator for Security and Counterterrorism, 2018 |
|  | The Dutch Program (unclear program name)  **Netherlands** | Yes | Yes  Offers aftercare | Reintegration  Disengagement | NPA | **Specific:**  -Trauma-informed approach  -Systems therapy  -Cognitive-behavioral therapy | | -Educational support | NPA | van der Heide, 2020 |
|  | The Norwegian Program;  **Norway** | Yes | Yes | Reintegration  Disengagement | Mandatory | **Specific:**  -Cognitive-behavioral therapy | | -Educational and vocational support  -Leisure activities | NPA | Hansen & Bjorgo, 2020 |
|  | Framework Program for Intervention in Violent Radicalization with Islamist Inmates;  **Spain** | Yes | Yes | Reintegration  Disengagement | Voluntary | Vague | | NPA | NPA | Garcia-Calvo & Vicente, 2020 |
|  | Desistance and Disengagement Program;  **United Kingdom** | Yes | Yes | Reintegration  Deradicalization  Disengagement | Mandatory | Vague | | -Vocational support  -Housing assistance  -General mentoring  -Religious counseling/ education/ mentoring | NPA | Weeks, 2021  Rosand, 2020  Basra, 2020  Smith, 2015 |
|  | Healthy Identity Intervention (HII);  **United Kingdom** | Yes | Yes and no | Disengagement | Voluntary | **Vague-informed approach**  -Mindfulness-based approach | | NPA | NPA | Basra, 2020  Smith, 2015 |
| North America | |  |  |  |  |  | |  |  |  |
|  | Evolve Program;  **Canada** | No | NPA | Reintegration  Disengagement | Voluntary | **Vague-informed approach**  -Trauma-informed approach | | -Assistance with basic needs, navigating bureaucratic  support systems, and accessing community resources  - Educational and vocational support  -Crisis management  -Religious counseling/ education/ mentoring | -Forensic Psychologists  -Social Workers  -Former extremists  -Islamic scholars | Organization for the Prevention of Violence, 2021  Evolve Program. (n.d.) |
|  | The Quebec Model;  **Canada** | Yes | No | Reintegration  Disengagement | NPA | **Vague-informed approach** Trauma-informed approach | | - Educational and vocational support  -Mentorship  -Artistic programs | -Psychiatrists  -Psychologists  -Social workers  -Psychoeducators | Rousseaau et al., 2022 |
| South America | |  |  |  |  |  | |  |  |  |
|  | Reincorporation Program;  **Columbia** | Yes | No | Reintegration | NPA  Must have disengaged prior | Vague | | -Health care  -Assistance with housing and other basic needs  -Vocational support | Social workers | Horgan & Braddock, 2009 |
| Australia and Oceania | |  |  |  |  |  | |  |  |  |
|  | PRISM;  **Australia** | Yes  Run by the Corrective Services of New South Wales | Yes | Reintegration  Disengagement | Voluntary | **Specific:**  -Motivational interviewing  -Cognitive-behavioral therapy | | Educational and vocational support  -Religious counseling/ education/ mentoring | -Psychologists  -Religious Support Officer  -Services and Programs Officers | Hassan et al., 2021  Zeuthen, 2021  Cherney & Belton, 2019  Cherney & Belton, 2020  Cherney, 2020 |
|  | Intervention 01;  **Australia** | Yes | NPA | NPA | NPA | Vague | | NPA | Police | Cherney, 2022 |

Note. The programs included in the table are those that were identifiable in the literature. It is possible other programs exist but have yet to be written about or are not yet accessible.

NPA: not publicly available

**Systematic Review References of the 66 sources**

**Southeast Asia Programs**

1. Alsubaie, B. A. (2017). Countering terrorism in the Kingdom of Saudi Arabia: An examination of the Prevention, Rehabilitation, and After-Care strategy (PRAC) [Doctoral dissertation, The University of New Haven]. Dissertations at the University of New Haven, 55.<https://digitalcommons.newhaven.edu/dissertations/55>
2. Bryans, S. (2016). *Handbook on the management of violent extremist prisoners and the prevention of radicalization to violence in prisons*. United Nations Office on Drugs and Crime. https://www.unodc.org/pdf/criminal_justice/Handbook_on_VEPs.pdf
3. El-Said, H. (2012). *De-radicalising Islamists: Programmes and their impact in Muslim majority states.* The International Centre for the Study of Radicalisation and Political Violence. https://icsr.info/wp-content/uploads/2012/02/1328200569ElSaidDeradicalisation1.pdf
4. Ezzarqui, L. (2010). De-radicalization and rehabilitation program: The case study of Saudi Arabia [Master’s thesis, Georgetown University]. Georgetown University.<http://hdl.handle.net/10822/553485>
5. Fink, N. C., & Hearne, E. B. (2008). *Beyond terrorism: Deradicalization and disengagement from violent extremism*. International Peace Institute. https://www.ipinst.org/wp-content/uploads/publications/beter.pdf
6. Gunaratna, R. (2021). Counter-terrorism in Asia: The state partnership with civil society organizations. In R. Gunaratna & M. M. Aslam (Eds.), *Civil Society Organizations Against Terrorism: Case Studies from Asia* (pp. 14-62). Routledge.<https://doi.org/10.4324/9781003150145>
7. Jayakumar, S. (2020). *Deradicalisation in Singapore: Past, present and future*. International Centre for the Study of Radicalisation. https://icsr.info/wp-content/uploads/2020/08/ICSR-Report-Deradicalisation-in-Singapore-Past-Present-and-Future.pdf
8. Mabborang, V. B., Jr. (2019). Assessing the effectiveness of the Philippines de-radicalization program [Master’s thesis, U.S. Army Command and General Staff College]. U.S. Army Command and General Staff College Fort Leavenworth United States. https://apps.dtic.mil/sti/pdfs/AD1105123.pdf
9. Milla, M. N., Hudiyana, J., & Arifin, H. H. (2020). Attitude toward rehabilitation as a key predictor for adopting alternative identities in deradicalization programs: An investigation of terrorist detainees’ profiles. *Asian Journal of Social Psychology, 23*(1), 15-28. https://doi.org/10.1111/ajsp.12380
10. Muluk, H., Umam, A. N., & Milla, M. N. (2020). Insights from a deradicalization program in Indonesian prisons: The potential benefits of psychological intervention prior to ideological discussion. *Asian Journal of Social Psychology, 23*, 42-53. https://doi.org/10.1111/ajsp.12392
11. Rabasa, A. Pettyjohn, S. L., Ghez, J. J., & Boucek, C. (2010). *Deradicalizing Islamist extremists.* Rand Corporation.<https://www.rand.org/pubs/monographs/MG1053.html>
12. Schenk, R. D., Griffin, S., Alia, M. L., & Stiglincová, K. (2021). *Complex terrorist cases for South and South-East Asia: Investigation, prosecution, adjudication, rehabilitation and reintegration*. United Nations Office on Drugs and Crime. <https://www.seanpve.org/Files/PublicationAttachments/PublicationAttachments_c899e4f0-3a12-4520-a5ad-d23664951a04.pdf>
13. Speckhard, A. (2011). Prison and community-based disengagement and de-radicalization programs for extremist involved in militant Jihadi terrorism ideologies and activities. In A. Speckhard (Ed.), *Psychosocial, organizational and cultural aspects of terrorism* (pp. 11.1-11.14). The Research and Technology Organisation of NATO. https://apps.dtic.mil/sti/pdfs/ADA555076.pdf

**South Asia Programs**

1. Basit, A. (2015). Pakistan’s militant rehabilitation programme: An overview. *Counter Terrorist Trends and Analysis, 7*(9), 10-17. https://www.jstor.org/stable/26351388
2. Basra, R. (2022). *Review of evidence: Prison-based interventions targeting violent extremist detainees.* Cross-Border Conflict Evidence, Policy and Trends. https://xcept-research.org/wp-content/uploads/2022/07/2022-07-19_RoE-Prison-Interventions-V4-Jul19.pdf
3. Bryans, S. (2016). *Handbook on the management of violent extremist prisoners and the prevention of radicalization to violence in prisons*. United Nations Office on Drugs and Crime. https://www.unodc.org/pdf/criminal_justice/Handbook_on_VEPs.pdf
4. Hassan, G., Brouillette-Alarie, S., Ousman, S., Savard, E. L., Kilinc, D., Madriaza, P., Varela, W., Pickup, D., Danis, E., the CPN-PREV team (2021). *A systematic review on the outcomes of tertiary prevention programs in the field of violent radicalization*. Canadian Practitioners Network for the Prevention of Radicalization and Extremist Violence. https://cpnprev.ca/systematicreview-3/
5. Hettiarachchi, M. (2013). Sri Lanka’s rehabilitation program: A new frontier in counter terrorism and counter insurgency. *PRISM, 4*(2), 105-121. https://www.jstor.org/stable/26469814
6. Hettiarachchi, M. (2018). Rehabilitation to deradicalise detainees and inmates: A counter-terrorism strategy. *Journal of Policing, Intelligence and Counter Terrorism, 13*(2), 267-283. https://doi.org/10.1080/18335330.2018.1476774
7. Hettiarachchi, M. (2019). Deradicalization of terrorist detainees and inmates: A soft approach to counter terrorism. In B. Schreer & A. T. H. Tan (Eds) *Terrorism and Insurgency in Asia: A Contemporary examination of terrorist and separatist movements* (pp. 214-230). Routledge. https://doi.org/10.4324/9780429031038
8. International Centre for Political Violence and Terrorism Research (ICPVTR) & S. Rajaratnam School of International Studies (RSIS) (2010a). *Project “Sabawoon”*. https://www.rsis.edu.sg/rsis-publication/icpvtr/report-1st-strategic-workshop-on-rehabilitation-and-de-radicalization-of-militants-and-extremist/?doing_wp_cron=1678809527.1085228919982910156250#.ZBCZvXbMK5c
9. International Centre for Political Violence and Terrorism Research (ICPVTR) & S. Rajaratnam School of International Studies (RSIS) (2010b). *Sri Lankan initiative in rehabilitation of former Tamil Tigers.* https://www.rsis.edu.sg/rsis-publication/icpvtr/report-1st-strategic-workshop-on-rehabilitation-and-de-radicalization-of-militants-and-extremist/?doing_wp_cron=1678809527.1085228919982910156250#.ZBCZvXbMK5c
10. Kapur, S. A. (2020). Bridging the gap between theory and praxis: An exploration of International society’s responsibility towards instability in Pakistan, 1947-2020 [Doctoral thesis, Lancaster University]. Lancaster University. https://doi.org/10.17635/lancaster/thesis/1196
11. Khan, S. E. (2015). *Deradicalization programming in Pakistan*. United States Institute of Peace. https://www.usip.org/publications/2015/09/deradicalization-programming-pakistan
12. Peracha, F., Khan, R. R., & Savage, S. (2016). Sabaoon: Educational methods successfully countering and preventing violent extremism. In S. Zeiger. (Ed.), *Expanding research on countering violent extremism* (pp. 85–104). Hedayah and Edith Cowan University.
13. Peracha, F., Savage, S., Khan, R. Ayub, A., & Zahra, A. (2022). Promoting cognitive complexity among violent extremist youth in northern Pakistan. *Journal of Strategic Security, 15*(1), 14-53. https://doi.org/10.5038/1944-0472.15.1.1943
14. Schenk, R. D., Griffin, S., Alia, M. L., & Stiglincová, K. (2021). *Complex terrorist cases for South and South-East Asia: Investigation, prosecution, adjudication, rehabilitation and reintegration*. United Nations Office on Drugs and Crime. <https://www.seanpve.org/Files/PublicationAttachments/PublicationAttachments_c899e4f0-3a12-4520-a5ad-d23664951a04.pdf>
15. Seifert, K. (2010). Can Jihadis be rehabilitated? Radical Islam. *Middle East Quarterly, (17)*2, 21-30. https://www.meforum.org/2660/can-jihadis-be-rehabilitated
16. Webber, D., Chernikova, M., Kruglanski, A. W., Gelfand, M. J., Hettiarachchi, M., Gunaratna, R., Lafreniere, M. A., & Belanger, J. J. (2018). Deradicalizing detained terrorists. *Political Psychology, 39*(3), 539-556. https://doi.org/10.1111/pops.12428

**Middle East Programs**

1. Alsubaie, B. A. (2017). Countering terrorism in the Kingdom of Saudi Arabia: An examination of the Prevention, Rehabilitation, and After-Care strategy (PRAC) [Doctoral dissertation, The University of New Haven]. Dissertations at the University of New Haven, 55.<https://digitalcommons.newhaven.edu/dissertations/55>
2. Anderlini, S. N., & Holmes, M. (2019). *Invisible women: Gendered dimensions of return, rehabilitation and reintegration from violent extremism*. International Civil Society Action Network. https://icanpeacework.org/2019/01/invisible-women/
3. Basra, R. (2022). *Review of evidence: Prison-based interventions targeting violent extremist detainees.* Cross-Border Conflict Evidence, Policy and Trends. https://xcept-research.org/wp-content/uploads/2022/07/2022-07-19_RoE-Prison-Interventions-V4-Jul19.pdf
4. Boucek, C. (2008a). Counter-terrorism from within: Assessing Saudi Arabia’s religious rehabilitation and disengagement programme. *RUSI Journal, 153*(6), 60-65. https://doi.org/10.1080/03071840802670106
5. Boucek, C. (2008b). Saudi Arabia’s “Soft” counterterrorism strategy: Prevention, rehabilitation, and aftercare, *Carnegie Papers, 97*, 1-28. https://carnegieendowment.org/files/cp97_boucek_saudi_final.pdf
6. Casptack, A. (2015). *Deradicalization programs in Saudi Arabia: A case study.* Middle East Institute. https://www.mei.edu/sites/default/files/Casptack.pdf
7. Davis, P., & Cragin, K. (Eds.). (2009). *Social science for counterterrorism putting the pieces together*. RAND Corporation. https://www.rand.org/pubs/monographs/MG849.html
8. El-Said, H. (2012). *De-radicalising Islamists: Programmes and their impact in Muslim majority states.* The International Centre for the Study of Radicalisation and Political Violence. https://icsr.info/wp-content/uploads/2012/02/1328200569ElSaidDeradicalisation1.pdf
9. Ezzarqui, L. (2010). De-radicalization and rehabilitation program: The case study of Saudi Arabia [Master’s thesis, Georgetown University]. Georgetown University.<http://hdl.handle.net/10822/553485>
10. Fink, N. C., & Hearne, E. B. (2008). *Beyond terrorism: Deradicalization and disengagement from violent extremism*. International Peace Institute. https://www.ipinst.org/wp-content/uploads/publications/beter.pdf
11. Hassan, G., Brouillette-Alarie, S., Ousman, S., Savard, E. L., Kilinc, D., Madriaza, P., Varela, W., Pickup, D., Danis, E., the CPN-PREV team (2021). *A systematic review on the outcomes of tertiary prevention programs in the field of violent radicalization*. Canadian Practitioners Network for the Prevention of Radicalization and Extremist Violence. https://cpnprev.ca/systematicreview-3/
12. Hill, G. (2016). Rehabilitating terrorists. *Journal of Eastern-European Criminal Law, 1*, 154-158.
13. Hoeft, G. (2015). *‘Soft’ approaches to counter-terrorism: An exploration of the benefits of deradicalization programs*. International Institute for Counter-Terrorism. https://www.ict.org.il/UserFiles/ICT-Soft-Approaches-to-CT-Hoeft.pdf
14. Horgan, J. & Braddock, K. (2009). *Assessing the effectiveness of current de-radicalization initiatives and identifying implications for the development of U.S.-based initiatives in multiple settings*. National Consortium for the Study of Terrorism and Response to Terrorism. https://www.start.umd.edu/research-projects/assessing-effectiveness-current-de-radicalization-initiatives-and-identifying
15. Horgan, J., & Braddock, K. (2010). Rehabilitating the terrorists?: Challenges in assessing the effectiveness of de-radicalization programs. *Terrorism and Political Violence, 22*(2), 267-291.<https://doi.org/10.1080/09546551003594748>
16. Rabasa, A. Pettyjohn, S. L., Ghez, J. J., & Boucek, C. (2010). *Deradicalizing Islamist extremists.* Rand Corporation.<https://www.rand.org/pubs/monographs/MG1053.html>
17. Speckhard, A. (2011). Prison and community-based disengagement and de-radicalization programs for extremist involved in militant Jihadi terrorism ideologies and activities. In A. Speckhard (Ed.), *Psychosocial, organizational and cultural aspects of terrorism* (pp. 11.1-11.14). The Research and Technology Organisation of NATO. https://apps.dtic.mil/sti/pdfs/ADA555076.pdf
18. Stern, J. (2010). Mind over martyr: How to deradicalize Islamist extremists. *Foreign Affairs, 89*(1), 95-108.
19. United Nations Office on Drugs and Crime (UNODC) (2021). *Technical assistance handbook on appropriate use of non-custodial measures for terrorism-related offenses.* United Nations Office on Drugs and Crime. https://www.unodc.org/pdf/terrorism/UNODC_Technical_Assistance_Handbook_-_Electronic_ENG.pdf

**Sub Saharan Africa Programs**

1. Anderlini, S. N., & Holmes, M. (2019). *Invisible women: Gendered dimensions of return, rehabilitation and reintegration from violent extremism*. International Civil Society Action Network. https://icanpeacework.org/2019/01/invisible-women/
2. Barkindo, A., & Bryans, S. (2016). De-radicalising prisoners in Nigeria: Developing a basic prison-based de-radicalisation programme. *Journal of Deradicalization*, 7, 1–25.
3. International Crisis Group (2021). An exit from Boko Haram? Assessing Nigeria’s operation safe corridor. *International Crisis Group*. http://www.jstor.org/stable/resrep31589
4. Hassan, G., Brouillette-Alarie, S., Ousman, S., Savard, E. L., Kilinc, D., Madriaza, P., Varela, W., Pickup, D., Danis, E., the CPN-PREV team (2021). *A systematic review on the outcomes of tertiary prevention programs in the field of violent radicalization*. Canadian Practitioners Network for the Prevention of Radicalization and Extremist Violence. https://cpnprev.ca/systematicreview-3/
5. Khalil, J. Brown, R. Chant, C. Olowo, P. & Wood, N. (2019). *Deradicalisation and disengagement in Somalia: Evidence from a rehabilitation programme for former members of Al-Shabaab*. Royal United Services Institute for Defence and Security Studies. https://rusi.org/explore-our-research/publications/whitehall-reports/deradicalisation-and-disengagement-somalia-evidence-rehabilitation-programme-former-members-al
6. Nemr, C., Nonninger, L., Entenmann, E., van Deventer, F., & van Ginkel, B. (2018). *An action agenda on the role of civil society in the rehabilitation and reintegration of those associated with and affected by violent extremism*. Global Center on Cooperative Security. https://www.icct.nl/publication/it-takes-village-action-agenda-role-civil-society-rehabilitation-and-reintegration
7. Onapajo, H., & Ozden, K. (2020). Non-military approach against terrorism in Nigeria: Deradicalization strategies and challenges in countering Boko Haram. *Security Journal 33*, 476-492.<https://doi.org/10.1057/s41284-020-00238-2>
8. Ugwueze, M. I., Ngwu, E. C., Onuoha, F. C. (2022). Operation Safe Corridor Programme and reintegration of ex-Boko Haram fighters in Nigeria. *Journal of Asian and African Studies, 57*(6), 1229-1248. https://doi.org/10.1177/00219096211047996
9. United Nations Office on Drugs and Crime (UNODC) (2021). *Technical assistance handbook on appropriate use of non-custodial measures for terrorism-related offenses.* United Nations Office on Drugs and Crime. https://www.unodc.org/pdf/terrorism/UNODC_Technical_Assistance_Handbook_-_Electronic_ENG.pdf
10. Zeuthen, M. (2021). *Reintegration: Disengaging violent extremists: A systematic literature review of effectiveness of counterterrorism and preventing countering violent extremism activities*. Royal United Services Institute.

**Europe Programs**

1. Basra, R. (2020). *Management in Europe: Country reports: Extremist offender management in England and Wales*. International Centre for the Study of Radicalisation. https://icsr.info/wp-content/uploads/2020/07/ICSR-Report-Extremist-Offender-Management-in-Europe-Country-Reports.pdf
2. Carlsson, L. (2021). *The role of psychotherapy in rehabilitation and exit work.* Radicalisation Awareness Network. https://home-affairs.ec.europa.eu/system/files/2021-11/ran_role_of_psychotherapy_in_rehabilitation_and_exit_work_112021_en.pdf
3. Christensen, T. W., & Bjorgo, T. (2018). *How to manage returned foreign fighters and other Syria travellers? Measures for safeguarding and follow-up*. C-REX Research Report.<https://www.sv.uio.no/c-rex/english/publications/c-rex-reports/2018/how-to-manage-foreign-fighters-c-rex-research-report.pdf>
4. Exit Deutschland (2014). *We provide the way out: De-radicalization and disengagement.* Exit Deutschland. https://www.exit-deutschland.de/english/
5. Fransen, R. (2021). *Reconstructing identity: The role of gender in driving and dismantling white supremacy in Sweden.* International Civil Society Action Network. https://icanpeacework.org/wp-content/uploads/2021/10/Sweden-Gender-and-Identity-Extremisms.pdf
6. Garcia-Calvo, C., & Vicente, A. (2020). *Management in Europe: Country reports: Extremist offender management in Spain*. International Centre for the Study of Radicalisation. https://icsr.info/wp-content/uploads/2020/07/ICSR-Report-Extremist-Offender-Management-in-Europe-Country-Reports.pdf
7. Hansen, D., & Bjorgo, T. (2020). *Management in Europe: Country reports: Extremist offender management in Norway*. International Centre for the Study of Radicalisation. https://icsr.info/wp-content/uploads/2020/07/ICSR-Report-Extremist-Offender-Management-in-Europe-Country-Reports.pdf
8. National Coordinator for Security and Counterterrorism (2018). *Evaluation of forsa and the family support centre.* National Coordinator for Security and Counterterrorism. https://www.landelijksteunpuntextremisme.nl/mediadepot/1702325ce56c/EvaluationofForsaandtheFamilySupportCentre.pdf
9. Radicalisation Awareness Network (RAN) (2019). *The disengagement/re-engagement path*. RAN Collection of Practices Search. https://home-affairs.ec.europa.eu/system/files/2022-10/the_disengagement_re-engagement_path_en.pdf
10. Renard, T. (2020). *Management in Europe: Country reports: Extremist offender management in Belgium*. International Centre for the Study of Radicalisation. https://icsr.info/wp-content/uploads/2020/07/ICSR-Report-Extremist-Offender-Management-in-Europe-Country-Reports.pdf
11. Rosand, E. (2020). *Non-custodial rehabilitation and reintegration in preventing and countering violent extremism and radicalization that lead to terrorism: A guidebook for policymakers and practitioners in south-eastern Europe*. Organization for Security and Cooperation in Europe. https://www.osce.org/secretariat/444838
12. Said, B. (2020). *Management in Europe: Country reports: Extremist offender management in Germany*. International Centre for the Study of Radicalisation. https://icsr.info/wp-content/uploads/2020/07/ICSR-Report-Extremist-Offender-Management-in-Europe-Country-Reports.pdf
13. Smith, A. (2015). *Radicalization and violent extremism: Lessons learned from Canada, the U.K. and the U.S*. National Institute of Justice. https://www.ojp.gov/pdffiles1/nij/249947.pdf
14. van der Heide, L. (2020). *Management in Europe: Country reports: Extremist offender management in Netherlands*. International Centre for the Study of Radicalisation. https://icsr.info/wp-content/uploads/2020/07/ICSR-Report-Extremist-Offender-Management-in-Europe-Country-Reports.pdf
15. Weeks, D. (2021). Lessons Learned from U.K. Efforts to Deradicalize Terror Offenders. *CTC Sentinal, 14*(3), 33-39. https://ctc.westpoint.edu/lessons-learned-from-u-k-efforts-to-deradicalize-terror-offenders/

**North America Programs**

1. Evolve Program (n.d.). *Frequently asked questions.* https://evolveprogram.ca/
2. Rousseau, C., Frounfelker, R., Ngov, C., & Crocker, A. (2022). Clinical services addressing violent extremism: The Quebec Model. *International Journal of Forensic Mental Health*, 1-11.<https://doi.org/10.1080/14999013.2022.2147254>
3. Organization for the Prevention of Violence (2021). *Evolve program: The first two years*. Organization for the Prevention of Violence. https://preventviolence.ca/publication/evolve-program-the-first-two-years/

**South America Programs**

1. Horgan, J. & Braddock, K. (2009). *Assessing the effectiveness of current de-radicalization initiatives and identifying implications for the development of U.S.-based initiatives in multiple settings*. National Consortium for the Study of Terrorism and Response to Terrorism. https://www.start.umd.edu/research-projects/assessing-effectiveness-current-de-radicalization-initiatives-and-identifying

**Australia and Oceania**

1. Cherney, A. (2020). Evaluating interventions to disengage extremist offenders: A study of the proactive integrated support model (PRISM). *Behavioral Sciences of Terrorism and Political Aggression, 12*(1), 17-36. https://doi.org/10.1080/19434472.2018.1495661
2. Cherney, A. (2022). Working with radicalised individuals: Insights from a secondary and tertiary prevention program. *Behavioral Sciences of Terrorism and Political Aggression*, 1-21.<https://doi.org/10.1080/19434472.2021.2013291>
3. Cherney, A., & Belton, E. (2019). Evaluating case-managed approaches to counter radicalization and violent extremism: An example of the proactive integrated support model (PRISM) Intervention, *Studies in Conflict & Terrorism, 44*(8), 625-645. https://doi.org/10.1080/1057610X.2019.1577016
4. Cherney, A., & Belton, E. (2020). Assessing intervention outcomes targeting radicalised offenders: Testing the pro integration model of extremist disengagement as an evaluate tool. *Dynamics of Asymmetric Conflict, 13*(3), 193-211.<https://doi.org/10.1080/17467586.2019.1680854>
5. Hassan, G., Brouillette-Alarie, S., Ousman, S., Savard, E. L., Kilinc, D., Madriaza, P., Varela, W., Pickup, D., Danis, E., the CPN-PREV team (2021). *A systematic review on the outcomes of tertiary prevention programs in the field of violent radicalization*. Canadian Practitioners Network for the Prevention of Radicalization and Extremist Violence. https://cpnprev.ca/systematicreview-3/
6. Zeuthen, M. (2021). *Reintegration: Disengaging violent extremists: A systematic literature review of effectiveness of counterterrorism and preventing countering violent extremism activities*. Royal United Services Institute.

1. Although interventions throughout Africa, South and Southeast Asia, and the Middle East clearly identify deradicalization as the program’s intended outcome, these interventions still require participants to disengage from violent extremist activity. In these programs, disengagement is *not* the intended outcome, but rather one of many strategies to facilitate deradicalization. [↑](#footnote-ref-1)
2. Within many Western programs, disengagement tends to be favored as an intended outcome over deradicalization, because it is perceived as a more realistic goal to achieve within the detention period (van der Heide, 2020). Nonetheless, many Western programs that do not target deradicalization explicitly still encourage individuals to critically reflect on their beliefs and discuss alternative perspectives. Such interventions include the Aarhus program in Denmark, the Forsa program in the Netherlands, the Healthy Identity Intervention (HII) in the United Kingdom, the PRISM intervention in Australia, the Swedish Entré program, and the Framework Program for Intervention in Violent Radicalization in Spain. [↑](#footnote-ref-2)
